# Aminoglycosides: Is there an effective alternative in severe tularaemia? A case series

**DOI:** 10.1007/s15010-026-02854-3

**Published:** 2026-07-15

**Authors:** Markus Fiedler, Matthias J. Neuböck, Johannes Eimer, Gregor Ablinger, Georg Murauer, Helmut J. F. Salzer, Thierry Rolling

**Affiliations:** 1 Department of Internal Medicine 4 - Pneumology, Kepler University Hospital, Linz, Austria; 2 Medical Faculty, Johannes Kepler University, Linz, Austria; 3 Division of Infectious Diseases and Tropical Medicine, Department of Internal Medicine 4 - Pneumology, Kepler University Hospital, Krankenhausstrasse 9, 4021 Linz, Austria; 4 Ignaz Semmelweis Institut, Interuniversity Institute for Infection Research, Vienna, Austria

**Keywords:** Fluoroquinolones, Aminoglycosides, Tularemia, Pulmonary tularemia

## Abstract

**Purpose:**

Severe pulmonary tularaemia is rare, and evidence guiding optimal antimicrobial treatment remains limited. Although aminoglycosides are commonly recommended, their toxicity profile and practical limitations may complicate treatments. The purpose of this case series was to describe clinical outcomes in adult patients with severe pulmonary tularaemia caused by *Francisella tularensis* who were treated with fluoroquinolones or tetracyclines.;

**Methods:**

We retrospectively describe six adult patients with severe pulmonary tularaemia. Clinical presentation, antimicrobial treatment, and clinical and radiological outcomes were reviewed, with a focus on response to targeted therapy with fluoroquinolones or tetracyclines.;

**Results:**

All six patients achieved complete clinical and radiological recovery following targeted antimicrobial therapy with either fluoroquinolones or tetracyclines despite severe pulmonary disease.;

**Conclusion:**

This case series provides consistent real-world evidence that fluoroquinolones and tetracyclines can achieve favourable outcomes in severe pulmonary tularaemia. These findings highlight their potential role as alternative treatment strategies and contribute meaningful data to the ongoing discussion on optimal antimicrobial management of tularaemia.

## Introduction

Tularaemia is a re-emerging zoonotic disease in Europe caused by the gram-negative bacterium *Francisella tularensis* [1], Transmission to humans most frequently occurs through direct contact with infected animals (notably hares and rodents), via arthropod vectors (especially ticks), ingestion of contaminated water or food, or, in fewer cases, by inhalation of infectious aerosols during activities like farming, hunting or landscaping [2]. Human to human transmission has not been reported [3].

Two major subspecies are relevant for Human-to-human infection: *F. tularensis* subspecies *tularensis* (Type A) and *F. tularensis* subsp. *holarctica* (Type B) [4]. In Europe, *F. tularensis* subsp*. holarctica* is the predominant strain, being less virulent, but widespread across Europe and Asia [5]. Outbreaks in Austria were rare and localized to regions like Upper- and Lower Austria, Burgenland, and Styria, often associated with hunting and handling of hares [1]. However, recent epidemiological evidence indicates spread to western provinces such as Salzburg, Tyrol, and Vorarlberg. Ticks, in Europe mainly *Ixodes ricinus,* are now recognized as essential vectors and the bacterium firmly established in much of Austria’s landscape [1, 6].

The clinical presentation of tularaemia varies depending on the route of infection, ranging from ulceroglandular and glandular forms to typhoidal and, importantly, pulmonary tularaemia. Pulmonary tularaemia is considered particularly severe owing to its atypical symptoms and diagnostic challenges. Patients may initially present with nonspecific symptoms such as fever, chills, malaise as well as respiratory symptoms including cough and dyspnoea, often resulting in misdiagnoses [7].

Severe tularaemia, including pulmonary forms, necessitates prompt and effective antimicrobial therapy. Aminoglycosides, such as gentamicin and streptomycin, are considered first-line agents for severe cases of tularaemia [5, 8]. More recently fluoroquinolones have been introduced as treatment options for severe disease.

We aim to provide additional clinical data on fluoroquinolone treatment of severe pulmonary disease and to describe outcomes from a recent series of tularaemia patients treated at our centre in Austria from December 2023 to December 2025.

## Case description

Details of the cases can be found in Table 1. All six patients with pulmonary tularaemia presented with an acute febrile illness with temperatures up to around 40 °C. Four patients maintained adequate oxygenation without supplemental support, whereas one patient required oxygen via nasal cannula and another was admitted to the intensive care unit for continuous positive airway pressure ventilation. All patients showed laboratory evidence of marked systemic inflammation (elevated C‑reactive protein). In some patients, additional organ involvement such as mild to moderate accompanying hepatitis with elevated hepatobiliary enzymes could be observed. No specific treatment was administered.

**Table 1:**
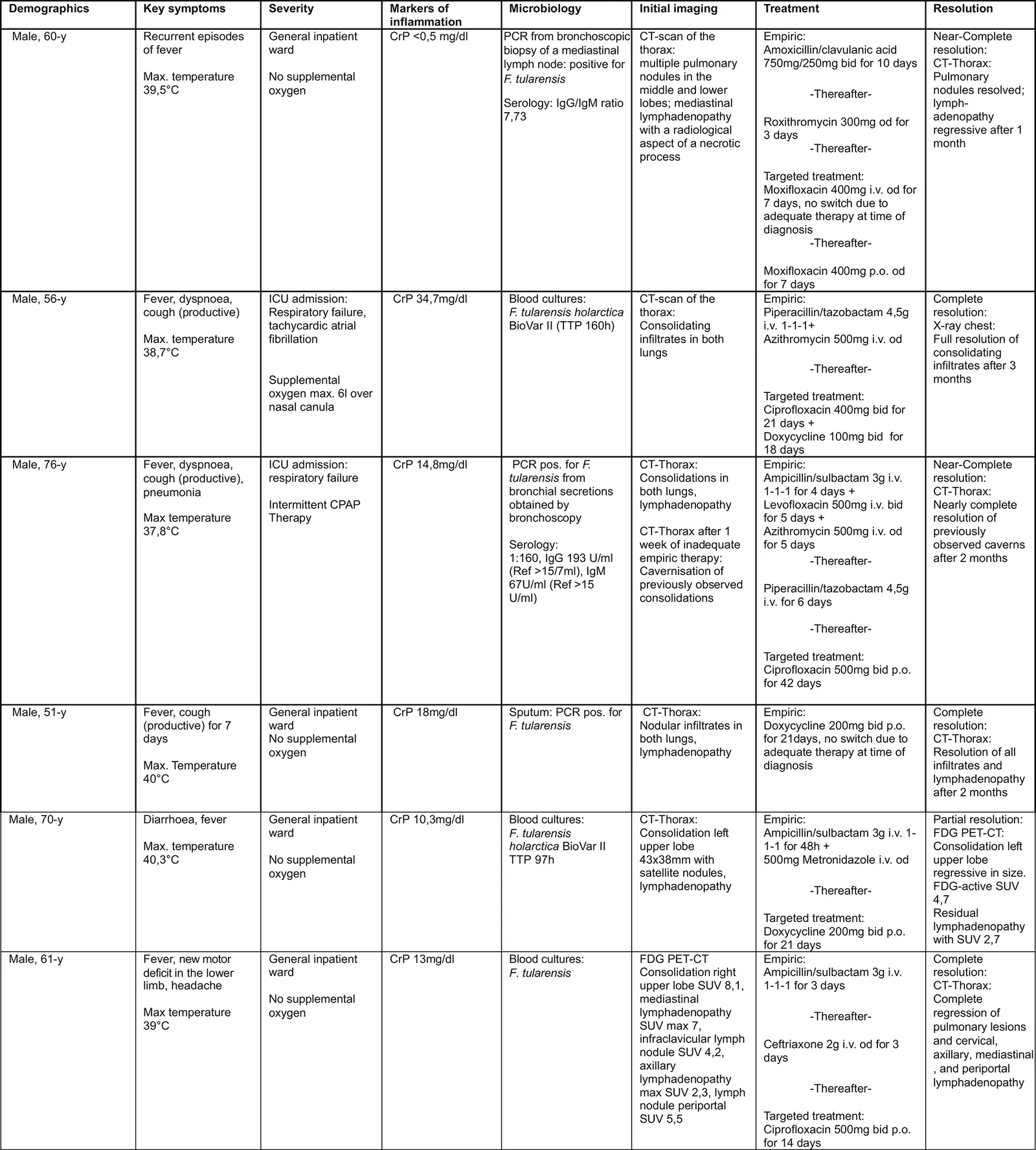
Clinical characteristics of the six adult patients with pulmonary tularaemia

Radiological imaging showed a pattern of consolidating or nodular pulmonary lesions with mediastinal lymphadenopathy. Additional lymph node involvement (cervical, axillary, periportal) and splenomegaly in several patients suggested systemic spread with predominant pulmonary involvement.

Further diagnostic tests included blood cultures in febrile patients as well as acquiring sputum samples. Furthermore, two patients underwent bronchoscopy where bronchial secretions was obtained. In one patient, a transbronchial needle-biopsy of an enlarged subcarinal lymph node was carried out.

Diagnosis was confirmed in all patients by direct microbiological evidence of *F. tularensis* subsp. *holarctica*, including polymerase chain reaction (PCR) from respiratory material or lymph node tissue and/or positive blood cultures, with additional serological support in some patients.

Five out of six patients initially received empiric broad-spectrum antimicrobials, which did not or only insufficiently improve the clinical and radiological picture, necessitating a switch to targeted therapy with agents active against *F. tularensis* once the pathogen was identified. Fluoroquinolone-based regimens (moxifloxacin or ciprofloxacin) and doxycycline, partly in combination, were administered for two to six weeks, initiated intravenously depending on disease severity and then continued orally. Treatment duration was depending on radiological pattern and resolution of the underlying lesions. Under this targeted treatment, all patients experienced a favourable course characterized by rapid defervescence, clinical improvement of respiratory symptoms, normalization of inflammatory and organ function parameters, and, over time, complete or near-complete resolution of pulmonary infiltrates, cavitations, and lymphadenopathy on imaging.

## Conclusion

All six described cases of severe pulmonary tularaemia support the evidence that treatment without aminoglycosides can be highly effective and highlight the importance of early pathogen-directed diagnostics. Each patient responded well to alternative regimens, most commonly fluoroquinolones (ciprofloxacin, moxifloxacin) and tetracyclines (doxycycline). One patient was switched to a combination of ciprofloxacin and doxycycline after three days of doxycycline monotherapy, due to a slow response with persistent fever. One patient was treated with moxifloxacin, leading to full resolution of symptoms, normalization of laboratory parameters, and radiological improvement in similar fashion compared to ciprofloxacin-based regimens. These outcomes indicate that even severe infections, including those requiring intensive care, can be successfully managed with non-aminoglycoside therapies when guided by targeted diagnostics and close clinical monitoring. Recent literature suggests that penetration into lung epithelial lining fluid by aminoglycosides is limited and characterised by pronounced interindividual variability when compared with corresponding serum concentrations [9]. Early bronchoalveolar-lavage investigations reported epithelial lining fluid levels of approximately 10–30% of contemporaneous plasma concentrations in the initial hours after intravenous administration, with wide between-patient dispersion [10]. More recent population pharmacokinetic analyses indicate that, despite attenuated peak concentrations, overall aminoglycoside exposure in epithelial lining fluid may attain roughly 50–100% of the plasma area under the concentration time curve owing to delayed equilibration into this compartment [9]. Collectively, these observations support the use of adequately high initial dosing and rigorous therapeutic drug monitoring in patients with severe pulmonary disease to ensure attainment of bactericidal concentrations at the pulmonary site of infection while minimising systemic toxicity [11]. Other options include fluoroquinolones (e.g., ciprofloxacin) and tetracyclines (e.g., doxycycline) where high concentrations in lung epithelial lining fluid can be observed [12, 13]. Despite the generally favourable prognosis with appropriate therapy, recurrence rates are not negligible, particularly, if initial treatment is delayed or incomplete [3]. CDC recommendations have been updated in October 2025 to support the use of fluoroquinolones and tetracyclines as first line antimicrobial agents [14]. Treatment decisions of cases reported in this case series was not influenced by this update, since the majority was diagnosed prior to the CDC´s publication. Our experience supports the use of fluoroquinolones and tetracyclines as viable alternatives for inpatient and outpatient management, avoiding the toxicity risks associated with aminoglycosides and strengthening the CDC´s position regarding the treatment of tularaemia. While ciprofloxacin has mostly been used as the quinolone of choice for tularaemia, we highlight a case of pulmonary tularaemia with good therapeutic response to moxifloxacin in line with the prior case series from Turkey on oropharyngeal and ulceroglandular tularaemia treated successfully with moxifloxacin [15, 16], and complementing the animal data on moxifloxacin for tularaemia indicated in the CDC guidelines [14]. As randomized clinical trials on tularaemia are unlikely due to its relative rarity, our case series adds to the evidence base in treating pulmonary tularaemia.

**Fig. 1:**
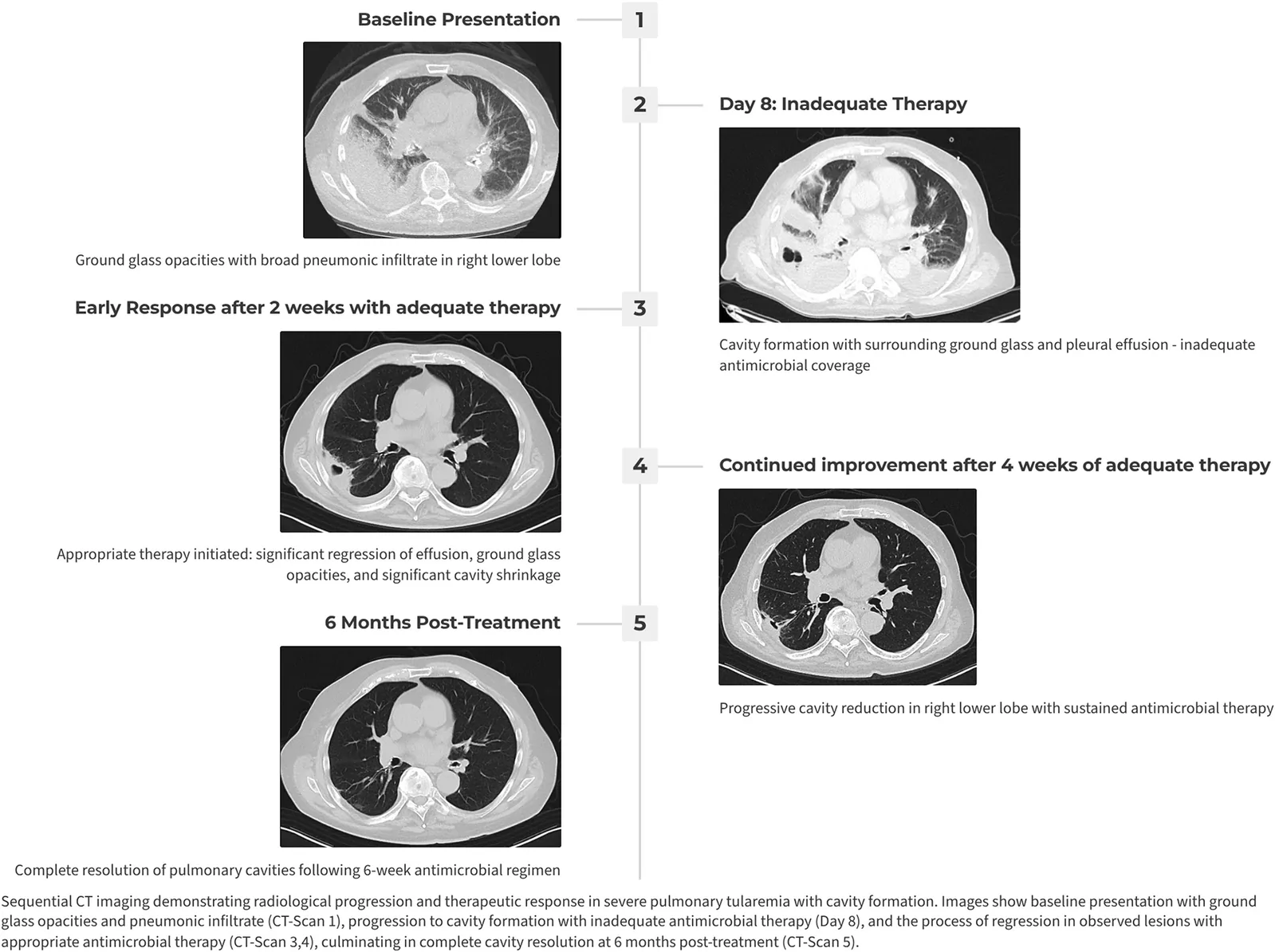
Clinical course: CT imaging timeline of a severe pulmonary tularaemia

## Data Availability

No datasets were generated or analysed during the current study.
